# Development of a blended learning system for nurses to learn the basics of psychoeducation for patients with mental disorders

**DOI:** 10.1186/s12912-021-00677-1

**Published:** 2021-09-09

**Authors:** Mitsunobu Matsuda, Ayumi Kohno

**Affiliations:** Psychiatric & Mental Health Nursing, Osaka City University Graduate School of Nursing, 1-5-17 Asahi-machi, Abeno-ku, 545-0051 Osaka, Japan

## Abstract

**Background:**

Psychoeducation should be practiced by various professionals. However, many Japanese psychiatric nurses recognize that psychoeducation should be practiced by other professionals, and show passive attitudes toward learning about evidence-based practices (EBPs), including psychoeducation. We developed a psychoeducation practitioner training program (PPTP) to nurture nurses. However, the PPTP was shown to be ineffective to help nurses achieve psychoeducation skills, although it improved their knowledge of psychoeducation and views on EBP. We developed and evaluated a revised version of the PPTP, integrating blended learning that combines e-learning and group education (BL-PPTP), to nurture nurses’ competencies to practice psychoeducation for patients with mental disorders.

**Method:**

We trained nurses working on acute psychiatric care wards of Japanese hospitals using BL-PPTP, and compared their attitudes for evidence-based practices (EBP attitudes), preparedness for psychoeducational practice, and self-efficacy at 4 points to clarify time-course changes in each participant.

**Results:**

Sixty-nine nurses participated, 31 withdrew, and 38 were analyzed. The time needed to complete BL-PPTP ranged from 31 to 259 days, revealing a marked individual difference. There were significant improvements in most participants’ EBP attitudes and preparedness for psychoeducational practice at the end of the program compared with the baseline.

**Conclusions:**

BL-PPTP may be useful to nurture nurses’ competencies to practice psychoeducation. BL-PPTP developed in the present study may also help disseminate psychoeducation among nurses, and increase the quality of nursing care.

## Background

In Japan, the mean length of stay in psychiatric hospitals is 262.4 days, which is much longer than those in other countries, and this is a challenge of its healthcare, medical, and welfare services for patients with mental disorders [1]. To address this, the government is establishing community-based care systems to support patients with mental disorders, placing importance on their community lives, rather than hospital treatment. However, the rates of rehospitalization within 6 and 12 months among discharged patients remain high, at about 30 and 37 %, respectively [2], indicating the necessity of discussing effective measures to help the patients continue their community lives. Japan may possibly be facing a situation similar to those of other countries, where poor medication adherence, insufficient family support, unfavorable interpersonal relationships, and stressful life events have been reported to lead to symptom recurrence and repeated hospitalizations [3].

In mental disorders, therapeutic effects are achieved only by integrating appropriate biological approaches, representing pharmacotherapy, and psychosocial approaches to maximize such effects and realize holistic support [4]. Japan is increasingly expanding community-based psychiatry, re-recognizing the importance of psychosocial approaches, and adopting measures to disseminate and promote them. As one of psychosocial approaches, psychoeducation for patients themselves has been reported to effectively improve their medication adherence [5, 6], recognition of pharmacotherapy [7, 8], and increase their quality of life (QOL) [6, 9].

However, psychoeducation for patients with mental disorders and their families has yet to be disseminated among Japanese psychiatric institutions. As barriers to the dissemination, organizational characteristics of these institutions, such as a lack of cooperative relationships with other departments, as well as factors related to staff’s recognition and skills, including an insufficient understanding of psychoeducation and scarcity of staff members with practical skills, have been noted [10]. Psychosocial approaches require skills to: establish empathetic and cooperative support relationships with patients with mental disorders; accurately clarify patients’ needs and set appropriate goals while promoting their voluntary participation; provide psychoeducation and Social Skills Training; and effectively use group works [11]. The establishment of education systems, covering supervision, is indispensable to train practitioners for these approaches [12], but neither methods for their education nor opportunities for such education are sufficiently available at present.

Psychoeducation should be practiced by various professionals. Among these professionals, nurses play an important role in collaborating with other professionals and connecting information from psychoeducation and patients’ experiences [13]. However, according to a survey on psychiatric nurses’ recognition in Japan [14], many nurses lack sufficient knowledge of psychoeducation and understanding of its effects. They show passive attitudes toward learning about evidence-based practices (EBPs), including psychoeducation. Thus, in order to disseminate psychoeducation, it may be necessary to convey the value of practicing it from the perspective of nursing to support patients’ daily lives, and promote such practice. Considering this, we previously developed an original 2-consecutive-day education program (Psychoeducational Practitioner Training Program: PPTP), consisting of lecture and training sessions to train psychoeducational practitioners [15]. PPTP increased participants’ knowledge of psychoeducation, sense of value related to it, and motivation to practice it, but it did not sufficiently help them acquire practical skills.

Psychoeducational practice requires extensive knowledge to cover a wide range of biological-psychological domains [16], in addition to advanced communication skills to manage patients’ narratives that vary according to situations. In this respect, we focused on the importance of promoting active learning and sincere attitudes toward daily nursing practice among individual nurses, and increasing their preparedness for the learning of methods to practice psychoeducation. We also regarded flexible styles essential to educate them while considering their learning achievement levels and work schedules. For these reasons, we used blended learning (BL) that combines web learning and group education to provide practical skill training.

 In the present study, we revised the previously developed PPTP, with BL incorporated, and evaluated the usefulness of the revised version, assuming that if it is shown to be useful, it may be applicable for psychoeducational practitioner training, and contribute to the dissemination of methods to practice psychoeducation among nurses engaged in healthcare, medical, and welfare services for patients with mental disorders.

Thus, the purposes of the present study were: to develop a revised version of PPTP using BL (BL-PPTP) that combines web learning and group education for nurses to acquire basic competencies to practice psychoeducation for patients with mental disorders; and to examine its usefulness, focusing on changes in nurses’ attitudes for EBPs (EBP attitudes), preparedness for psychoeducational practice, and self-efficacy.

## Materials and methods

### Outline of BL-PPTP

BL-PPTP consisted of (1) web learning to learn psychoeducation [17] and (2) group education to acquire practical skills for psychoeducation (Table 1). To systematize learning, only participants who had completed web learning were allowed to participate in group education. Before the start of learning, a login password required for web learning was allocated to each nurse who had returned a consent form.

**Table 1:** Contents of BL-PPTP

**Web learnig**	
**Original Textbook**	
Chapter 1: Basics of Psychoeducation	
Chapter 2: Outline of NPE	
Chapter 3: Basic Knowledge of NPE	
Chapter 4: Practice of NPE	
**Original DVD**	
Showing simulated psychoeducation practice	
**Group education for practical skill training**	
**Group structure**	
5-7 participants/group	
**Time**	
6 hours/day	
**Methods**	
Participants played nurse and patient roles	
Participants confirm their own performance during practical skill training using videos	
Participants obtain opinions and advice from other participants and BL-PPTP developers	

### Web Learning

The concept of web learning was defined as allowing participants to: (1) easily use the system, (2) participate individually, (3) resume learning at any time after interruption, (4) record the date and time of each session, (5) understand the ground for each content, (6) comprehend each term, and (7) visualize sessions of psychoeducation. For web learning, a textbook created based on < A Nurse-led Version of Psychoeducation Program for Patients with Schizophrenia, Referred to as Nursing Psychoeducation (NPE)> [18], which had been used for the PPTP lecture, and original DVD that contains a video of psychoeducational practice simulation were used. The NPE is a four-session intervention program whose goal is to improve acceptance of medication and illness of patients with schizophrenia, and to improve their medication adherence in acute psychiatric units (Table 2). Two psychiatric nurses (leader, co-leader) who trained in NPE conducted the intervention.

**Table 2:** Contents of NPE

**Time**	60-90 minutes/day, a day/week, total 4 days.
**Group structure**	A closed group with 5-7 participants
**Method**	Information based on textbook & sharing of experience
**Learning materials** (Textbook)	1. Types of symptoms of psychogenic illness
	2. Association between psychogenic illness and stress
	3. Primary effect and side effect of medication
	4. How to adjust to living with illness in the community

The textbook consists of [Chapter 1: Basics of Psychoeducation], [Chapter 2: Outline of NPE], [Chapter 3: Basic Knowledge of NPE], and [Chapter 4: Practice of NPE], and learning goals in each chapter were set and explained in detail. The DVD plays back several practical NPE sessions, with the NPE developer as the leader, and 1 and 5 psychiatric nurses with experience of NPE practice in clinical settings as the co-leader and patients, respectively. The duration of each session after editing is approximately 30 min. The web learning system was accessible from personal computers and smartphones. It was designed to help learners achieve a certain level of knowledge by adding the following functions: (1) presenting explanatory notes when learners click technical terms, (2) conducting a mini-test with 10–20 questions to be answered by selecting from multiple choices or entering correct words before advancing to the next chapter, (3) defining a correct answer rate of 70 % as the requirement for passing, and not allowing those not meeting this requirement to advance to the next chapter, and (4) randomly presenting test questions.

### Group Education for Practical Skill Training

The concept of group education was defined as allowing participants to: (1) view role-playing and simulations of psychoeducation and (2) receive feedback from others. Practical skill training consisted of role-play and reflection using a feedback support tool (PF-NOTEv2, Photron) [19]. During role-play, participants acted as the leader, co-leader, or patients based on PPTP contents for practical skill training, and the roles were rotated, so that participants could experience all of them. The group education was provided by a developer of Nursing Psychoeducation. For effective skill acquisition, appropriate learning environments, where participants can confirm their own performance during practical skill training using videos, in addition to obtaining opinions and advice from others, are required [20]. In communication skill training, feedback from others promotes awareness of non-verbal communication styles [21]. Therefore, we used a feedback support tool that instantly records/plays back videos for reflection for participants to visually confirm their performance during training. After each session, all participants watched a video of practical skill training, evaluated each other, listing good points and points to be improved, and repeated training.

### Participants

The participants were nurses working on acute psychiatric care wards of psychiatric hospitals in the Kinki and Tokyo areas of Japan. For the selection of hospitals, the welfare and medical service network system WAM NET was used. A letter of request for participation and poster for public relations activities were first mailed to the nursing directors of candidate psychiatric hospitals. Subsequently, copies of a letter of recruitment and document explaining the procedure to participate were sent to consenting hospitals, asking to distribute this document among nurses working on the relevant wards. Nurses’ declarations to participate in the study were collected by e-mail, and then a consent form was mailed to each nurse, asking them to sign and return the form by mail.

### Data Collection

To collect data, a series of surveys were conducted using a structured questionnaire. In addition to participants’ characteristics: the age, sex, lengths of nursing and psychiatric nursing experiences (years), qualification, position, times needed to complete the entire BL-PPTP and web learning only (days), and period between the end of web learning and start of group education (days), and scores from 3 assessment scales were examined. An approximately 15-minute survey was conducted at 4 points: (1) immediately before the start of web learning (Time 1), (2) immediately after the final test for web learning (Time 2), (3) immediately before group education (Time 3), and (4) immediately after group education (Time 4). Surveys 1 and 2 were conducted as part of web learning, and 3 and 4 were conducted at the group education venue using sheets.

### Measurements

For assessment, 2 scales with sufficient reliability and validity and an original questionnaire to measure preparedness for psychoeducational practice were used.

### EBP Attitudes

For this assessment, we used the Japanese Version of the Evidence-Based Practice Attitude Scale (EBPAS-J) [22]. This scale is a modified Japanese version by Okumura et al., which was originally developed by Aarons GA [23], and statistically examines its reliability and validity. In the present study, we adopted this version to measure EBP attitudes. EBPAS measures attitudes toward new treatments and interventions and views on their usability. There are 15 statements representing 4 subscales: openness to autonomously implement EBPs (< Openness>), willingness to implement required EBPs (< Requirements>), intuitive appeal of EBPs (< Appeal>), and attitudes toward not implementing EBPs in clinical settings (< Divergence>: reverse item), which are rated on a 5-point Likert scale from “Strongly disagree” to “Strongly agree”. Higher scores indicate more positive attitudes toward implementing EBPs. In the present study, Cronbach’s alpha was 0.39 for the entire scale, 0.55 for < Openness>, 0.40 for < Appeal>, 0.61 for < Requirements>, and 0.11 for < Divergence>.

### Self-efficacy

For this assessment, we used the General Self-efficacy Scale (GSES) [24]. This scale measures self-efficacy, which refers to the belief in one’s capabilities to organize and execute behaviors required to produce specific performance achievements, and it consists of 16 statements to be rated on a 2-point scale (“Yes”/“No”). Higher scores indicate higher levels of self-efficacy. In the present study, Cronbach’s alpha for the entire scale was 0.32.

### Preparedness for Psychoeducational Practice

For this assessment, we used an original questionnaire to measure knowledge, emotions, sense of value, motivation, and skills to practice psychoeducation (Psychoeducation Preparedness Questionnaire: PPQ) based on a definition of clinical nursing competences by Defloor et al. [25]. The questionnaire consisted of statements, which were created through a review of the literature on competencies to practice psychoeducation [15, 18, 26–29] and brainstorming by researchers with an experience of 10 years or longer in psychoeducation. Subsequently, to ensure sufficient content validity, appropriate statements were selected from those created and categorized upon deliberations among researchers well-versed in psychoeducation. Consequently, 28 statements representing the following 4 categories were adopted: <understanding of and respect for patients>: e.g., understanding the characteristics of each disorder and waiting or drawing out their narratives; <sense of value related to psychoeducational practice and motivation for it>: e.g., reducing patients’ anxiety after discharge and looking for QOL improvement; <communication skills>: e.g., concentrating on patients’ narratives, utilizing nonlinguistic communication, and becoming aware of the necessity of improving one’s own communication skills; and < concerns related to psychoeducational practice> (reverse item): e.g., lacking sufficient knowledge and skills for psychoeducation and being concerned over barriers to establishing psychoeducation in the facility. Each statement was rated on a 4-point Likert scale from “Strongly agree” to “Strongly disagree”, and higher scores indicate higher levels of preparedness for psychoeducational practice. In the present study, Cronbach’s alpha was 0.84 for the entire scale, 0.83 for < understanding of and respect for patients>, 0.71 for < valuing psychoeducation and being motivated to practice it>, 0.81 for < communication skills>, and 0.85 for < concerns over psychoeducational practice>.

### Analysis

We evaluated BL-PPTP by comparing the results of the 4 surveys, with Survey 1 as the baseline, and analyzing time-course changes in each participant. Prior to analysis, we conducted the Shapiro-Wilk normality test to confirm the normality of all data. Time-course changes were examined through repeated measure analysis of variance and multiple comparisons adopting the Bonferroni method. All analytical processes were performed using SPSS 26.0 for Windows.

## Results

### Participants’ Characteristics

Sixty-nine nurses participated, 17 withdrew during web learning, and 14 completed web learning, but they did not participate in group education. Thus, the withdrawal rate was 44.93 %. The reasons for withdrawal from web learning were unclear, but the most frequent reason for not participating in group education was “None of the group education sessions are convenient”. The number of participants finally included for analysis was 38 (55.07 %). Their attributes were as follows: mean age: 39.79 (SD = 7.33); sex: male: 17 (44.74 %), female: 21 (55.26 %); mean length of nursing experience: 12.76 (8.45) years; mean length of psychiatric nursing experience: 9.22 (6.99) years; qualification: nurse: 37 (97.37 %), 1 (2.63 %); position: staff nurse: 27 (71.05 %), chief nurse: 6 (15.79 %), nursing director: 4 (10.53 %), and N/A: 1 (2.63 %).

On the other hand, the times needed to complete the entire BL program and web learning only ranged from 31 to 259 (mean = 92.3, SD = 45.6, median = 76.5) and from 1 to 144 (29.4, 30.2, 17.0) days, respectively. The period between the end of web learning and start of group education ranged from 3 to 239 (64.0, 38.7, 55.5) days (Table 3).

**Table 3:** Participants' characteristics of the study sample (*N*=38)

	n (%) or mean ± SD
Age mean	39.79 ± 7.33
Gender	
Male	17 (44.74)
Female	21 (55.26)
Mean length of nursing experience (Years)	12.76 ± 8.45
Mean length of psychiatric nursing experience (Years)	9.22 ± 6.99
Qualification	
Registered nurse	37 (97.37)
Assistant nurse	1 (2.63)
Position	
Staff nurse	27 (71.05)
Chief nurse	6 (15.79)
Nursing director	4 (10.53)
N/A	1 (2.63)
Times needed to complete the entire BL-PPTP (Days)	92.34 ± 45.64
Times needed to complete the web learning (Days)	29.37 ± 30.19
Period between the end of web learning and start of group education (Days)	63.97 ± 38.67

### Assessment Scale Scores

The results of analysis of assessment scale scores are presented in Table 4. On comparing EBPAS-J scores before and after BL-PPTP, significant differences were observed in the total score and scores for < Openness>, <Divergence>, and < Appeal> (*p* < 0.000). Multiple comparisons revealed that the total score was significantly higher in Times 2–4 compared with the baseline. It was also significantly higher in Time 4 compared with 2 and 3. As for the subscales, <Openness > scores were significantly higher in Times 2–4 compared with the baseline. They were also significantly higher in Time 4 compared with 2 and 3. <Divergence > scores were significantly higher in Times 2–4 compared with the baseline. Moreover, <Appeal > scores were significantly higher in Times 2–4 compared with the baseline. They were also significantly higher in Time 4 compared with 2 and 3, whereas there were no significant differences in < Requirements > scores (*p* = 0.273).

**Table 4:** Time-course changes of EBPAS-J, GSES and PPQ in participants (*N*=38)

	Before web learning (Time 1)		After web learning (Time 2)		Before group education (Time 3)		After group education (Time 4)		F-test		Bonferroni
	mean	SD	mean	SD	mean	SD	mean	SD	F-value	*P*-value	
**EBPAS-J**											
total score	32.00	4.55	40.92	6.07	40.29	5.20	44.34	6.75	64.16	0.000	Time 1 < 2 Time 1 < 3 Time 1 < 4 Time 2 < 4 Time 3 < 4
Openness	1.49	0.52	2.35	0.81	2.21	0.62	2.73	0.71	40.41	0.000	Time 1 < 2 Time 1 < 3 Time 1 < 4 Time 2 < 4 Time 3 < 4
Divergence	2.38	0.50	3.24	0.49	3.38	0.46	3.34	0.56	53.22	0.000	Time 1 < 2 Time 1 < 3 Time 1 < 4
Appeal	2.32	0.63	2.84	0.64	2.78	0.61	3.11	0.69	20.83	0.000	Time 1 < 2 Time 1 < 3 Time 1 < 4 Time 2 < 4 Time 3 < 4
Requirements	2.40	0.69	2.39	0.86	2.28	0.76	2.54	0.95	1.32	0.273	
**GSES**											
Standaraized score	45.58	5.52	42.68	10.49	41.24	11.00	41.84	11.33	2.67	0.097	
**PPQ**											
Total score	74.82	8.16	76.50	7.00	73.13	7.75	78.00	9.22	7.04	0.001	Time 2 > 3 Time 3 < 4
Understanding of and respect for patients	21.95	3.37	21.74	2.25	21.11	2.67	22.29	2.96	2.80	0.043	Time 3 < 4
Sense of value related to psychoeducational practice and motivation for it	18.55	2.21	19.61	2.48	19.37	2.11	21.03	2.20	14.71	0.000	Time 1 < 4 Time 2 < 4 Time 3 < 4
Communication skills	25.50	3.83	27.16	2.67	25.95	3.32	27.66	3.66	7.65	0.000	Time 1 < 2 Time 1 < 4 Time 2 > 3 Time 3 < 4
Concerns related to psychoeducational practice	8.82	3.24	8.00	2.36	6.71	2.45	7.03	2.78	6.51	0.003	Time 1 > 3 Time 2 > 3 Time 2 > 4

As for PPQ, there were significant differences in the total score and scores for all categories between before and after BL-PPTP (*p* < 0.05). Multiple comparisons revealed that the total score was significantly lower in Time 3 compared with 2, but it was significantly higher in Time 4 compared with 3. Scores for < understanding of and respect for patients > were significantly higher in Time 4 compared with 3. Scores for < valuing psychoeducation and being motivated to practice it > were significantly higher in Time 4 compared with the baseline, Time 2, and Time 3. Scores for < communication skills > were significantly higher in Times 2 and 4 compared with the baseline, and in Time 4 compared with 3, but they were significantly lower in Time 3 compared with 2. Lastly, scores for < concerns over psychoeducational practice > were significantly lower in Time 3 compared with the baseline and in Times 3 and 4 compared with 2. No significant differences were observed in GSES scores (*p* = 0.097).

## Discussion

### Participants’ Characteristics

The participants’ mean age was 39.79. Males and females accounted for 44.74 and 55.26 %, respectively. The mean length of nursing experience was 12.76 ± 8.45 (psychiatric nursing: 9.22 ± 6.99) years. As these characteristics are similar to those of participants of a workshop for psychiatric nurses in Japan (mean age: 40.8, male: 36.0 %, female: 64.0 %, and length of nursing experience: 17.9 years) [30], it is reasonable to regard the subjects of the present study as an average group.

The time needed to complete BL-PPTP ranged from approximately 1 to 9 months. The time needed to complete web learning ranged from 1 day to approximately 5 months. The period between the end of web learning and start of group education ranged from 3 days to approximately 8 months. Thus, there were marked individual differences. As a background factor, it should be noted that 94.74 % of the participants were staff nurses on acute psychiatric care wards. During the program, they may have learned in their own styles at their own paces while working shifts. This supports BL-PPTP, placing importance on learning attitudes and methods, as an educational approach that allows participants to learn in their own styles at their own paces.

On the other hand, the withdrawal rate was high, and the sample size was small in the present study. It was 2000, when the first report on nursing education based on computer-assisted instruction (CAI) [31] was made in Japan, and the number of nursing educational institutions actively promoting CAI-based learning is still limited. Based on this, the participants may have been inexperienced at web learning, and they as novice psychoeducation learners may have found BL-PPTP difficult in terms of learning content and system manipulation. The advantage of web learning is allowing learners to participate in their free time anytime and anywhere, but such flexibility may result in procrastination, delayed progress, or interruption as a disadvantage. Therefore, it may be necessary to establish systems to field questions that arise during web learning, and create opportunities for participants to communicate with each other, in order to help them continue learning.

### Usefulness of the Program

In the present study, we aimed to develop BL-PPTP, and confirm its usefulness. On assessing the participants’ EBP attitudes, significant improvements were observed in the total score and scores for < Openness>, <Divergence>, and < Appeal>. Such improvements were also observed in the 4 categories presenting preparedness for psychoeducational practice, as scores were generally higher after group education than at the baseline. On the other hand, the mean GSES score gradually decreased, although the difference was not significant.

The mean scores for < Openness > and < Appeal > as subscales representing EBP attitudes generally improved with progress in learning. BL-PPTP facilitates systematic learning, combining web learning to theoretically learn psychoeducation and practical skill training to comprehensively learn the theory and practice. In BL-PPTP, participants’ attitudes toward the application of psychoeducation in actual settings and perception of its appeal may be promoted through web learning and practical skill training as part of group education. However, the lower mean score before the start of group education compared with that at the end of web learning suggests a correlation with the period between the end of web learning and start of group education and temporarily reduced awareness and motivation.

Scores for < Divergence>, representing attitudes toward not implementing EBPs in clinical settings, significantly increased with progress in learning. As this subscale is a reverse item, the participants possibly revised their attitudes through repeated learning. Thus, by participating in BL-PPTP, they may have learned the depths of psychoeducation and communication skills that are indispensable for its management, and consequently become more motivated to practice psychoeducation as an EBP.

Scores for < understanding of and respect for patients > as a PPQ category did not markedly change after web learning, but they increased through practical skill training as part of group education, indicating that web learning was not effective to promote the participants’ attitudes for understanding and respecting patients, whereas practical skill training using a feedback support tool helped them visually evaluate their own performance during training, and obtain third-party advice, leading to improvements in these aspects. As for < valuing psychoeducation and being motivated to practice it>, scores generally improved through learning in BL-PPTP. In addition to extensive knowledge to cover a wide range of biological-psychological domains [32], skills to listen/speak to others, clearly and understandably communicate [33], and develop interactions with others, adopting techniques of SST/group therapy [34] are also required in psychoeducational practice. As BL-PPTP is designed to help individual participants first autonomously acquire basic knowledge of psychoeducation, and watch psychoeducation sessions video-recorded in a DVD utilizing a web learning system, and then practice what they have previously learned in group education, it may have been useful for the participants to stably develop a sense of value related to psychoeducational practice. Scores for < communication skills > significantly improved at the ends of web learning and group education compared with the baseline, but they temporarily decreased before the start of group education. Such a decrease may also be explained by the period between the end of web learning and start of group education, and it is likely that the participants subsequently restored their self-confidence, and improved their communication skills through practical skill training.

In daily nursing practice, nurses develop appropriate communication while protecting patients’ dignity. However, on some occasions, their statements and behaviors may be judged as inappropriate by others. In the present study, practical skill training using a feedback support tool may have provided opportunities for nurses to observe their own performance during training in real time, visually understand appropriate statements and behaviors and those to be improved in each situation, and perform reflection. It may also have helped them improve their own thoughts, accepting advice from other participants and instructors, and acquire skills, including those used for group therapy, which are difficult to convey in other settings than practice [35].

As a negative result, scores for < concerns over psychoeducational practice > increased with progress in learning. This may be explained by participants’ anxiety and concerns about a lack of sufficient skills to practice psychoeducation in their facilities, similar to the case of the EBPAS-J subscale < Divergence>. However, it may be more reasonable to consider that such concerns may be resolved through future continuous training than negatively taking them, as it is likely that the participants simply became determined to practice psychoeducation only after acquiring sufficient skills, in order to prevent the disbenefit of patients due to insufficient practice. Lastly, the lack of improvement in self-efficacy reveals the difficulty of increasing self-efficacy as an individual trait only through knowledge and skill acquisition. At the same time, it suggests that although the time needed to complete BL-PPTP ranged from 31 to 259 days, and some participants took a long time to complete it, this did not negatively affect their self-efficacy.

### Practice Implications

The present study may have novelty in examining methods for practitioner training toward the dissemination of psychoeducation, as few studies have addressed this topic. To help patients with mental disorders comfortably live in their communities, not only support for medication adherence, but also support for overall QOL improvement is indispensable. In this respect, nurses’ learning of methods for psychoeducational practice is important to increase the quality of nursing care. The BL-PPTP developed in the present study may be useful to disseminate psychoeducation among nurses, and increase the quality of nursing care.

### Limitations and Future Research

The present study has the following limitations: First, the high withdrawal rate and small sample size may have influenced the in- and external validity. Second, the developed BL-PPTP nurtured nurses’ basic competencies to practice psychoeducation, but we did not examine the durability of this effect. Therefore, we have 3 challenges to address in future studies: (1) increasing the sample size, and further examining the study items, (2) reducing the withdrawal rate by reviewing the difficulty level we set for web learning, and helping participants maintain their motivation to learn, and (3) conducting follow-up to help participants further improve their skills and maintain their motivation for psychoeducational practice. Finally, as a strategy to implement BL-PPTP, it may be effective to actively provide those in charge of the management of psychiatric facilities, such as facility managers and directors of nursing, with psychoeducation-related information and encourage their participation in it.

## Conclusions

We created a revised-version of PPTP, integrating BL that combines web learning and group education, and evaluated its usefulness. BL-PPTP did not increase participants’ self-efficacy, but it improved their EBP attitudes, as the total score and scores for < Openness>, <Divergence>, and < Appeal > significantly improved. Participants generally became more prepared for psychoeducational practice after group education compared with the baseline. The results support the usefulness of BL-PPTP to nurture nurses’ basic competencies to practice psychoeducation.

## Data Availability

The datasets used and/or analyzed during the current study are available from the corresponding author on reasonable request.

## Abbreviations

QOL: Quality of Life
EBPs: Evidence-Based Practices
PPTP: Psychoeducational Practitioner Training Program
BL: Blended Learning
BL-PPTP: Psychoeducational Practitioner Training Program using Blended Learning
